# Evolutionary history predicts the response of tree species to forest loss: A case study in peninsular Spain

**DOI:** 10.1371/journal.pone.0204365

**Published:** 2018-09-20

**Authors:** Rafael Molina-Venegas, Sonia Llorente-Culebras, Paloma Ruiz-Benito, Miguel A. Rodríguez

**Affiliations:** Departamento de Ciencias de la Vida, Universidad de Alcalá, Madrid, Spain; Fred Hutchinson Cancer Research Center, UNITED STATES

## Abstract

Evolutionary history can explain species resemblance to a large extent. Thus, if closely related species share combinations of traits that modulate their response to environmental changes, then phylogeny could predict species sensitivity to novel stressors such as increased levels of deforestation. To test this hypothesis, we used 66,949 plots (25-m-radius) of the Spanish National Forest Inventory and modelled the relationships between local (plot-level) stem density of 61 Holarctic tree species and forest canopy cover measured at local and landscape scales (concentric circles centred on the plots with radiuses of 1.6, 3.2 and 6.4 km, respectively). Then, we used the output model equations to estimate the probability of occurrence of the species as a function of forest canopy cover (i.e. response to forest loss), and quantified the phylogenetic signal in their responses using a molecular phylogeny. Most species showed a lower probability of occurrence when forest canopy cover in the plots (local scale) was low. However, the probability of occurrence of many species increased when forest canopy cover decreased across landscape scales. We detected a strong phylogenetic signal in species response to forest loss at local and small landscape (1.6 km) scales. However, phylogenetic signal was weak and non-significant at intermediate (3.2 km) and large (6.4 km) landscape scales. Our results suggest that phylogenetic information could be used to prioritize forested areas for conservation, since evolutionary history may largely determine species response to forest loss. As such, phylogenetically diverse forests might ensure contrasted responses to deforestation, and thus less abrupt reductions in the abundances of the constituent species.

## Introduction

Deforestation is one of the most ubiquitous threats to biodiversity [1], notably affecting species interaction networks [2], driving population shrinking [3], and ultimately leading to species extinction [4]. Further, forest loss may alter a disparate set of ecosystem processes such as nutrient cycling and carbon fluxes [5], thus endangering ecosystem services on which human well-being depends [6].

The most immediate effect of forest reduction is habitat loss for many forest-affiliated species, yet species inhabiting open habitats may benefit from forest-cover reduction [7, 8]. Although the effects of forest loss on individual species are rather complex [9, 10], there is increasing evidence that species response to forest loss is tightly linked to their functional and life-history traits [7, 11, 12], and thus we may expect a certain degree of interdependence in their response as far as they show some phenotypic resemblance [13]. Identifying the specific combinations of functional traits that best predict species response to forest loss–i.e. the so-called deforestation-sensitivity syndromes [14]–might be useful to diagnosing extinction risk in threatened forest ecosystems [15] and informing conservation planning.

The use of functional traits to study species response to forest loss has been possible thanks to the increased effort to collect a wide spectrum of traits from different taxonomic groups. However, even one of the largest and most comprehensive functional trait databases compiled to date (i.e. the TRY database for plants [16]) is largely incomplete for many traits and taxonomic groups. For example, specific leaf area is a well-known trait that has been used in continental-scale tree studies [17], yet it is recorded for only 3.5% of global plant diversity and generally biased towards frequently measured species [18]. Therefore, trait information may be too sparse and patched to detect deforestation-sensitivity syndromes, particularly in species-rich ecosystems and for largely uncharacterized taxa (e.g. terrestrial invertebrates [19]). Moreover, evidence of deforestation-sensitivity syndromes in the literature is sometimes weak [20] or even contradictory (see [12] and references therein), which might reflect the difficulties inherent to identifying and measuring functionally relevant traits for species response to forest loss [21].

Besides functional traits, the availability of phylogenetic information across multiple taxonomic groups is increasing exponentially [22–25]. As such, phylogenetic data may represent a powerful alternative to functional trait-based approaches for devising early diagnosis of biodiversity vulnerability to forest loss. If phylogenetic signal in the combinations of functional traits that modulate species responses to deforestation pressures is high (i.e. evolutionary conservatism in deforestation-sensitivity syndromes [26]), close relatives would tend to show similar responses to forest loss [12], and thus species vulnerabilities to forest reduction could be anticipated from phylogenetic affiliations.

Forest loss is the immediate consequence of deforestation, yet the spatial configuration of the remaining forest fragments in the landscape may have also important implications for biodiversity [27]. As such, some species may be favoured in landscapes made up of small and isolated forest patches due to e.g. positive edge effects [28, 29, 8] or density compensation (i.e. release of competition due to extinction of competitors that are negatively affected by fragmentation [30]). Although there is evidence that forest loss has a stronger effect on biodiversity than the spatial configuration of forest fragments in the landscape ([31], but see [32]), the relative importance of both processes is still under debate [33, 34], likely mirroring the inherent difficulties to tease apart their specific effects with observational data (but see [29, 8]).

**Fig 1:**
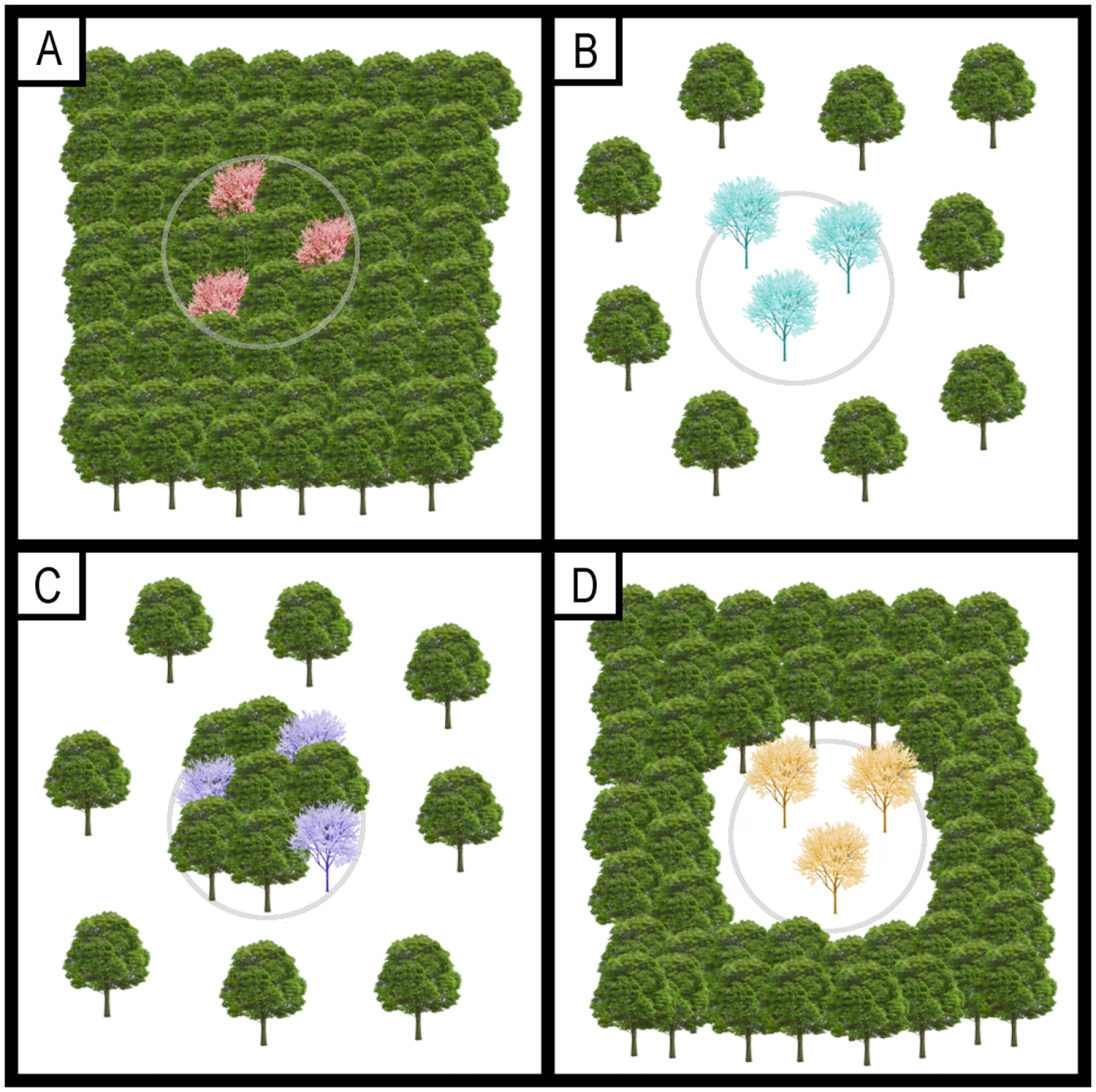
Conceptual model depicting the response of tree species to plot-level (local) and landscape forest canopy cover. The colored trees represent individuals of the target species, and the grey circumferences represent the borders of the sampling units (i.e. circular plots) that are placed across the landscape. (A) The species responds negatively to forest loss at either local (plot-level) and landscape scales (the entire area within the top-left panel), and thus it only occurs in extensively forested areas. (B) The species responds positively to forest loss at both scales, and therefore it tends to grow in extensively deforested areas. (C) The species responds negatively to forest loss at the local scale, but positively at the landscape scale. As such, it tends to occur in isolated forest fragments. (D) The target species responds positively to forest loss at the local scale, but negatively at the landscape scale, meaning that it is an early successional species that regenerates mainly within forest gaps.

In this study, we used 66,949 circular plots (25-m-radius) of the third Spanish National Forest Inventory to model the relationships between the local stem density of 61 Holarctic tree species and the percentage of forest canopy cover measured in the plots (local scale) and in the neighbouring landscape (i.e. concentric circles centred on the plots with radiuses of 1.6, 3.2 and 6.4 km, respectively). Then, we applied the model outputs along with phylogenetic information to estimate: (i) the response of tree species to forest loss (i.e. species probability of occurrence as a function of forest canopy cover), and (ii) the phylogenetic signal in species responses. The relationship between the probability of occurrence of species and plot-level canopy cover would serve to detect species degree of forest affiliation (i.e. species preferences for closed or more open forest canopies), whereas the relationship between the probability of occurrence and canopy cover in the neighbouring landscape would indirectly reflect species response to the spatial configuration of forest fragments. For instance, a forest-affiliated species will show a high probability of occurrence when plot canopy cover is high. However, the same species may show a low probability of occurrence when canopy cover in the neighbouring landscape is also high, suggesting that the species is prone to occur in isolated forest patches (Fig 1c). In contrast, a species that shows a high probability of occurrence when canopy cover is high at both scales will tend to form continuous and extensive forest masses (Fig 1a).

## Materials and methods

### Study region

**Fig 2:**
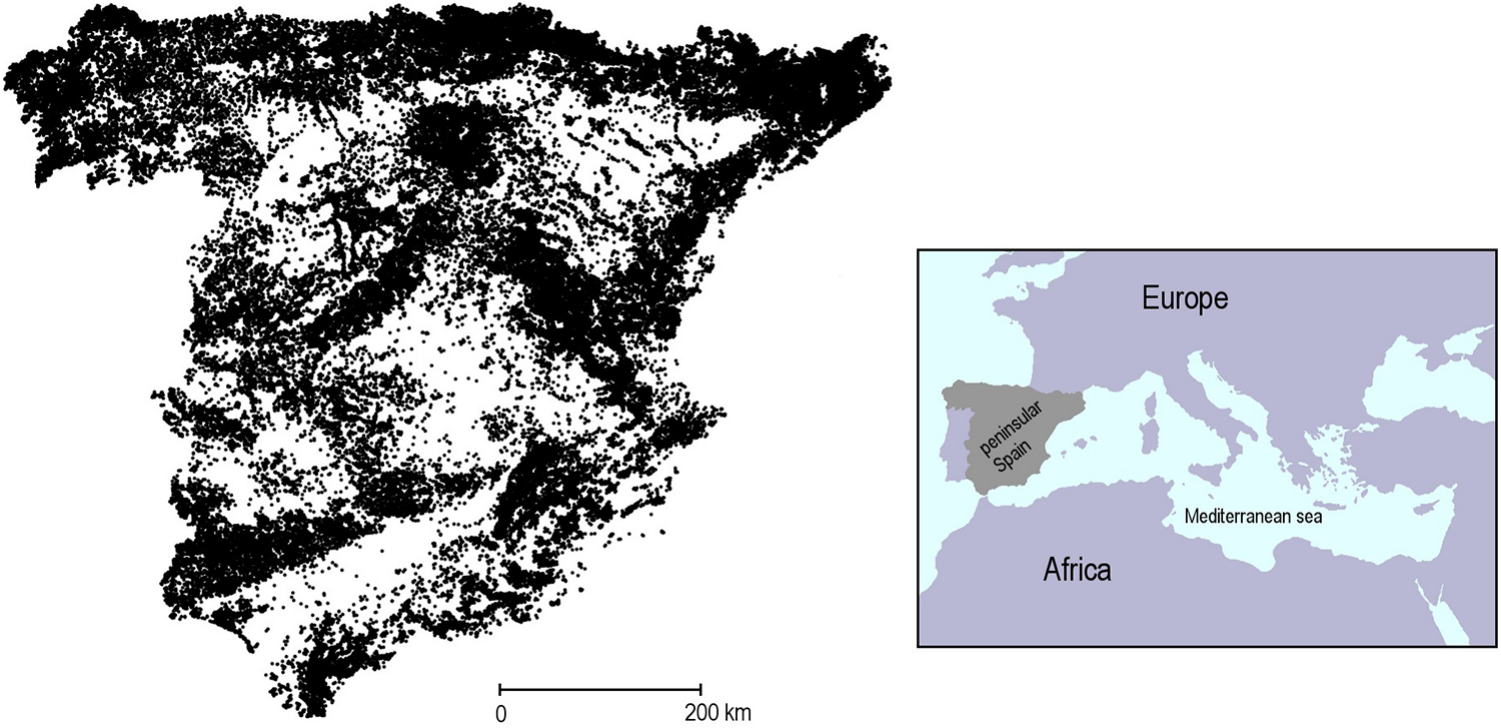
Distribution of survey plots in peninsular Spain. In total, *n* = 66,949 circular plots of 25-m-radius were analysed. The inset shows the location of the study area (shaded area) in the western edge of the Mediterranean.

Peninsular Spain is located in the Iberian Peninsula at the southwest corner of Europe (Fig 2). The region possesses a high physiographic complexity, with several mountain ranges running, primarily, east-west. The northern third of peninsular Spain (i.e. Galician Massif, Cantabrian Mountains and western and central Pyrenees) falls within the Eurosiberian biogeographic zone, and it is characterized by a humid climate that is moderated by the influence of the Atlantic Ocean. Winters are relatively cold and there is lack of drought season (precipitation ranges from approximately 1000 mm to more than 1500 mm). Roughly, the vegetation is deciduous oak dominated forest at valley bottoms (i.e. with *Quercus petrea*, *Q*. *robur* and *Fraxinus excelsior* among others), with beech (*Fagus sylvatica*) and fir (*Abies alba*) forests at intermediate and high elevations, respectively [35]. Birches (*Betula pendula*) often constitute small enclaves in the clearings of the beech forest, and the mountain pine (*Pinus uncinata*) forms the characteristic subalpine natural forest in the Pyrenees.

The rest of peninsular Spain is dominated by the Mediterranean climate, with relatively soft winters and a marked summer drought (precipitation ranges from less than 350 mm to 1500 mm). The typical forests in this region include evergreen trees such as holm oak (*Quercus ilex*), Portuguese oak (*Q*. *faginea*), cork oak (*Q*. *suber*), Pyrenean oak (*Q*. *pyrenaica*), Algerian oak (*Q*. *canariensis*), juniper (*Juniperus sp*.) and wild olive (*Olea europaea*) among others. These are accompanied or replaced in the warmer or steeper regions by forests of Aleppo pine (*Pinus halepensis*) and in areas of sandy ground by the stone pine (*P*. *pinea*). The Scots pine (*P*. *sylvestris*) also forms extensive forests in the mountainous areas of the northern half of peninsular Spain.

### Forest data

First, we compiled a list of tree species from the third Spanish National Forest Inventory (IFN3) [36]. We did not include species of hybrid origin, nor infra-specific taxa, and conducted standardization of nomenclatural criteria by means of The Plant List [37]. Then, we removed species that are strictly cultivated in the study area according to Castroviejo [38] and those that occurred in less than 25 plots of the IFN3 (to avoid low statistical-power issues). Finally, we retained the species that matched the phylogenetic tips of the phylogeny used in the analyses (see below). This procedure resulted in a list of 61 Holarctic tree species (46 angiosperms and 15 gymnosperms).

**Fig 3:**
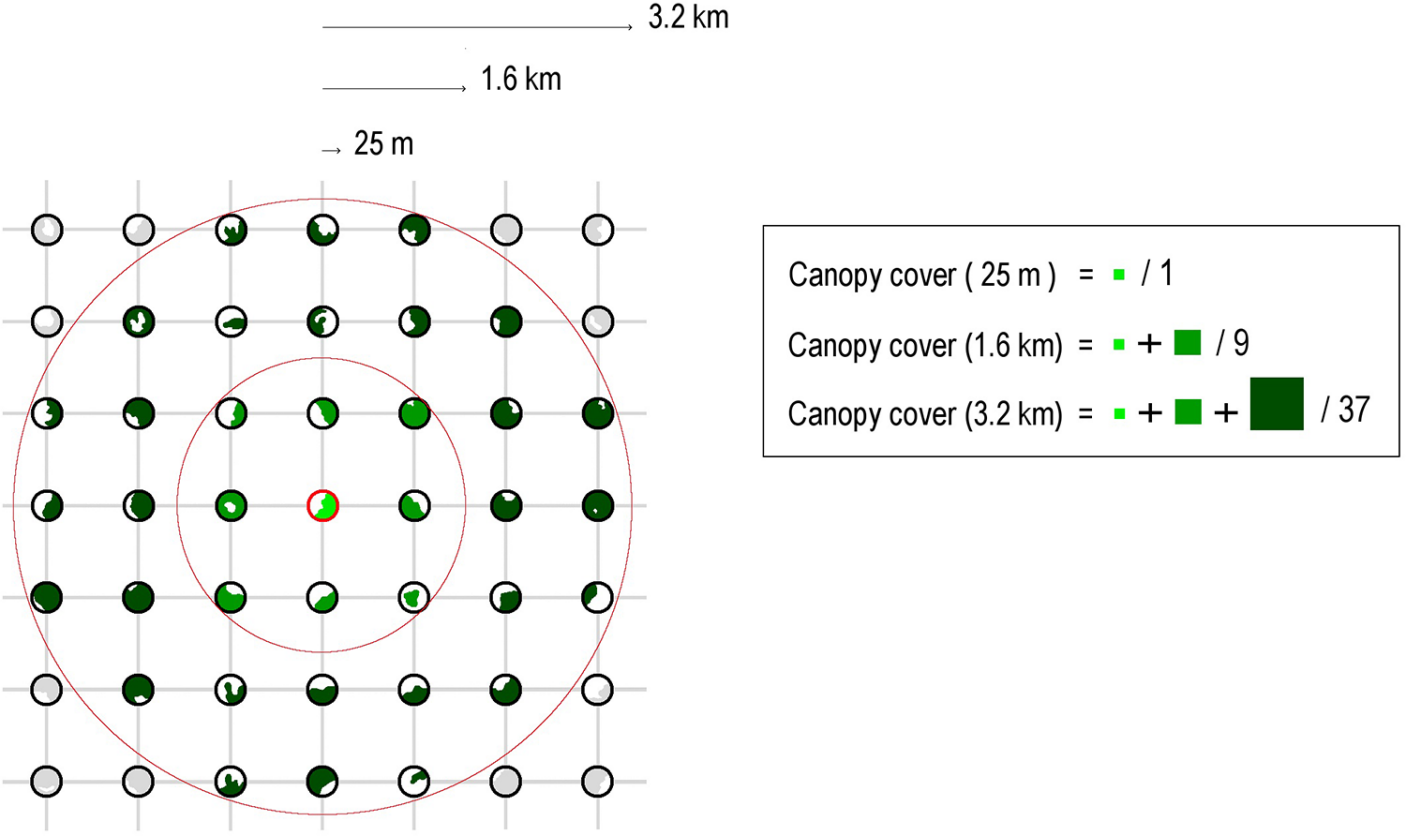
Schematic representation of the sampling method used to measure forest canopy cover at different spatial scales. Circular plots of 25-m-radius were placed in the intersections of a 1-km grid that encompassed the study area (peninsular Spain). The percentage of forest canopy cover (green-coloured areas) was measured within each plot (local scale) and at three different spatial scales in the neighbouring landscape, which was represented by three concentric circles centred on the plots with radiuses of 1.6, 3.2 and 6.4 km, respectively (for simplicity, only the local and the 1.6 and 3.2 km landscape scales are represented). Canopy cover was averaged from the maximum number of plots that each circle could possibly hold. The 25-m-radius circles are drawn out of the scale of the grid (1 km) to facilitate visualization of the plots. The legend schematically summarizes the calculations for estimating forest canopy cover in a given 25-m-radius plot (in red) and in the neighbouring landscape of the target plot (only 1.6 and 3.2 km-radius circles are shown).

We used 66,949 IFN3 survey plots distributed across peninsular Spain in which the focal species occurred (Fig 2). The IFN3 placed a circular sample plot of 25-m-radius in the intersections of a 1-km grid that were classified as forested by the Forest Map of Spain (MAPAMA—MFE50; available at http://www.mapama.gob.es). From the IFN3, we extracted two different variables measured in the plots: (i) the stem density of each species (i.e. individuals larger than 1.30 m of height and 75 mm of diameter at breast height per hectare), and (ii) the percentage of forest canopy cover (i.e. projected area of adult trees canopy), which was estimated “de visu” [36]. Besides, we also estimated forest canopy cover at three different spatial scales in the neighbouring landscape of the plots, which was represented in this case by three concentric circles centred on the plots with radiuses of 1.6, 3.2 and 6.4 km, respectively. To do so, we averaged the canopy cover recorded in the plots that each circle could possibly hold (i.e. n = 9, 37 and 129, respectively; Fig 3), thus representing forest canopy cover in three different landscape buffer areas. Because borders with Portugal and France do not represent natural barriers to forest establishment, we excluded all plots located less than 6.4 km apart from them. In sum, our dataset consisted in a local estimate of stem density for each species (response variable) and one local and three landscape-level estimates of forest canopy cover (explanatory variables).

### Phylogenetic tree

We used a species-level time-calibrated molecular phylogeny including native tree species (woody plants growing to ≥ 4 m) of Europe and North America that was pruned to retain only the 61 species of our study (see [39] for details on the phylogenetic procedure). The phylogeny was inferred with maximum-likelihood methods based on a mixed supertree-supermatrix approach [40], with sequences corresponding to various chloroplastic (i.e. rbcL, matK, trnL-F) and nuclear DNA regions (i.e. ITS1 and ITS2). The maximum likelihood tree obtained in the phylogenetic inference analysis was calibrated using the software TreePL [41]. To do so, we used 14 calibration points (fossil data) with minimum and maximum age constraints extracted from [42] and [43] (S1 Table). The age of the root node (Euphyllophyta) was fixed at 365 ma [44]. The smoothing parameter *ρ* that affects the penalty for rate variation over the tree was estimated using cross-validation (here, *ρ* = 0.1).

### Statistical analyses

#### Response to canopy cover

We fitted Poisson models to explore the relationship between the probability of occurrence of each species and forest canopy cover [45]. The Poisson distribution describes the probability of a given number of events (i.e. in our study, the observed stem density of each species in the plots where they occurred) in a fixed interval of the explanatory variable (i.e. forest canopy cover, in our study):

$$P\left(\rho_{j},F_{q}\right)=\frac{\lambda^{\rho \text{j}}*e^{-\lambda }}{\rho_{j}!}$$ (1)

$$\lambda \left(j,\;F_{q}\right)=M_{j}+(C_{j}*F_{q})$$ (2)

where ρ_*j*_ is the stem density of species *j* in plot *q*; *F*_*q*_ represents canopy cover in plot *q* (or alternatively the canopy cover in the neighbouring landscape of plot *q*); λ is the rate parameter of the Poisson distribution, which here represents the expected stem density for species *j* along the canopy cover gradient; e is the base of the natural logarithm; and *M*_*j*_ and *C*_*j*_ are species-specific parameters for species *j* and with respect to *F*_*q*_. Then, when the probability *P* of presence of species *j* is zero, *P* only depends on the rate parameter (λ):

$$P\left(\rho_{j}=0,F_{q}\right)=e^{-\lambda }$$ (3)

And we can transform Eq 3 as follows:

$$P\left(\rho_{j}>0,\;F_{q}\right)=1-e^{-\lambda }$$ (4)

where *P* in Eq 4 is the probability of finding species *j* along the canopy cover gradient irrespective of the stem density of species *j* in plot *q*, which allows to explore the relationship between the species probability of occurrence and forest canopy cover. For each species and spatial scale, we evaluated the informative power of the models (i.e. probability of finding species *j* as a function of forest canopy cover) with respect to the corresponding intercept models using the Akaike Information Criterion (i.e. AIC_c_). Thus, we conducted 244 independent pairwise model comparisons (i.e. *n* = 61 species × 4 spatial scales), and assumed that forest canopy cover was a good predictor of species presence when the AIC_c_ difference between pairwise models was higher than 10 [46]. Otherwise we did not consider the models in subsequent analyses. To evaluate which spatial scale best described species presence, we also compared the models fitted for each species across the different spatial scales (i.e. four models per species) using AIC_c_.

In order to facilitate comparison among species we used the fitted models (only those that showed an AIC_c_ > 10 with respect to the corresponding intercept models) to estimate, for each species *j*, a scalar Ω_*j*_ defined as the natural logarithm of the ratio between occurrence probabilities at a low and a high level of forest canopy cover; i.e. 5 and 75% respectively (see [29] for a similar analysis):

$${Ω}_{j}=\text{ln}\frac{P\left(j,\;F=5\%\right)}{P\left(j,\;F=75\%\right)}$$ (5)

Positive Ω values imply that the species are more likely to be found under open forest canopies (i.e. positive association between forest loss and species abundances), while negative Ω values indicate the opposite. For each species, we estimated one Ω_*j*_ value at the local scale (using plot canopy cover as the explanatory variable) and three Ω_*j*_ values at the landscape scales (i.e. using the averaged canopy cover of each concentric circle centred on the target plot, Fig 3). To test the robustness of estimates and plausible values for the true Ω_*j*_, we generated non-parametric 95% confidence intervals (95% CI) using a bootstrap procedure [29]. We produced 1000 bootstrapped samples for each species *j* by resampling with replacement from the original sample of *j* (the bootstrapped samples comprised the same number of plots as the original samples), and used the bootstrapped samples to parametrize the Poisson model and compute 1000 Ω values (Eq 5). The positions 25^th^ and 976^th^ of the ranked values correspond to the lower and upper limits of 95% CI. We considered species responses to decreased canopy cover were non-neutral (i.e. negative or positive) when their corresponding CI did not include zero [7].

#### Phylogenetic signal in species response to forest loss

Phylogenetic signal can be defined as the degree of statistical dependence among species trait values due to their phylogenetic relationships [47, 48]. As such, high phylogenetic signal indicates that ancestor-descendant relationships explain a high fraction of the variance in a certain trait (e.g. Ω values), whereas low phylogenetic signal indicates the opposite (i.e. the trait has evolved following uncorrelated trajectories). In order to measure phylogenetic signal in species response to forest loss (i.e. Ω values) we used the Pagel’s λ statistic [49]. Pagel’s λ is ranged between 0 (complete lack of phylogenetic signal) and 1 (the trait has evolved following a pure Brownian motion model of evolution). Unlike other commonly used indices of phylogenetic signal (e.g. Blomberg’s *K*), Pagel’s λ is robust to phylogenetic resolution and branch-length information uncertainties [50, 51]. The statistical significance of λ was assessed based on a likelihood ratio test as implemented in the *phylosig* function of phytools R package [52].

To account for the uncertainty associated to Ω values in phylogenetic signal estimation, we created a dataset of Ω trait values (*n* = 1000) for each species *j* by randomly sampling from the uniform distribution U_*j*_ (min Ω_*j*_, max Ω_*j*_), where min Ω_*j*_ and max Ω_*j*_ are minimum and maximum values of the 95% CI. We obtained a distribution of λ values and their associated *p*-values from the so-generated Ω traits (*n* = 1000 tests), and reported the overall phylogenetic signal (λ statistic) and statistical significance (*p*-value) based on the median value of the 1000 iterations. We conducted the analyses using either all species in the phylogeny and angiosperms and gymnosperms, separately.

## Results

**Fig 4:**
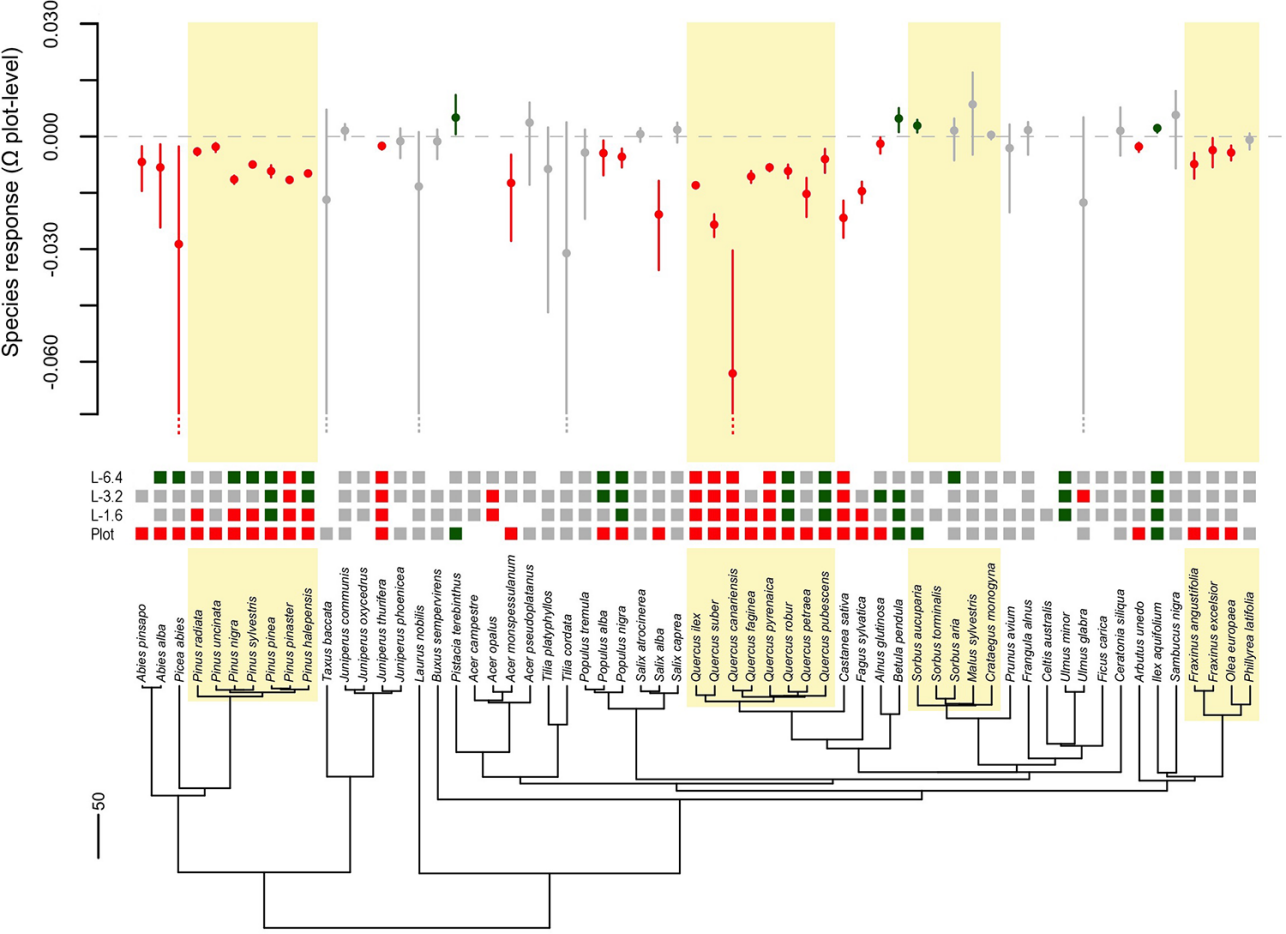
Response of tree species (Ω values) to forest canopy cover measured in the plots (local scale) and in the neighbouring landscape (L-1.6, L-3.2 and L-6.4). The squared symbols on the phylogenetic tips show the type of response of each species (negative, neutral or positive), and the bars and dots correspond to 95% CI of Ω values estimated at the plot level. The response of species was considered negative when the 95% CI of Ω completely laid below zero (red colour), positive when the 95% CI completely laid above zero (green colour), and neutral if the 95% CI included zero (grey colour). The gaps in the figure represent those cases where the Poisson models failed to explain the probability of occurrences of species (see text). The scale bar in the phylogeny represents millions of years. The highlighted clades include species that showed similar responses to plot-level canopy cover within their respective lineages.

At the plot scale, canopy cover was a good predictor of species stem density in 89% of the cases (i.e. 54 out of 61 tree species). Of this pool, 30 species (56%) showed consistently negative responses to decreased canopy cover (i.e. negative Ω values with confidence intervals not including zero), four species (7%) showed consistently positive responses (i.e. positive Ω values with confidence intervals not including zero), and 20 species (37%) showed a neutral response (i.e. either positive or negative Ω values with confidence intervals including zero, Fig 4). Overall, most of the species that had a negative response to decreased forest canopy cover at the plot scale showed a neutral or positive response at the landscape scales (Figs 4 and 2c). However, some species showed consistently negative responses across all scales, most of them within the Fagaceae (Figs 4 and 2a), and a few species showed positive responses across most of the scales analysed (Figs 4 and 2b). No species showed positive response at the plot scale but negative at the landscape scales (Figs 4 and 2d). The robustness of estimates of Ω values was overall high (i.e. narrow 95% CI), yet a few species showed substantially wide confidence intervals (Fig 4 and S1 Fig). We found that in 59% of the cases (36 out of 61 species analysed), forest canopy cover at the local scale was the best predictor of species presence (i.e. lowest AIC_c_ score), whereas canopy cover at the 1.6, 3.2 and 6.4-km-radius scales were the best predictors for 18%, 10% and 13% of the species analysed, respectively.

**Fig 5:**
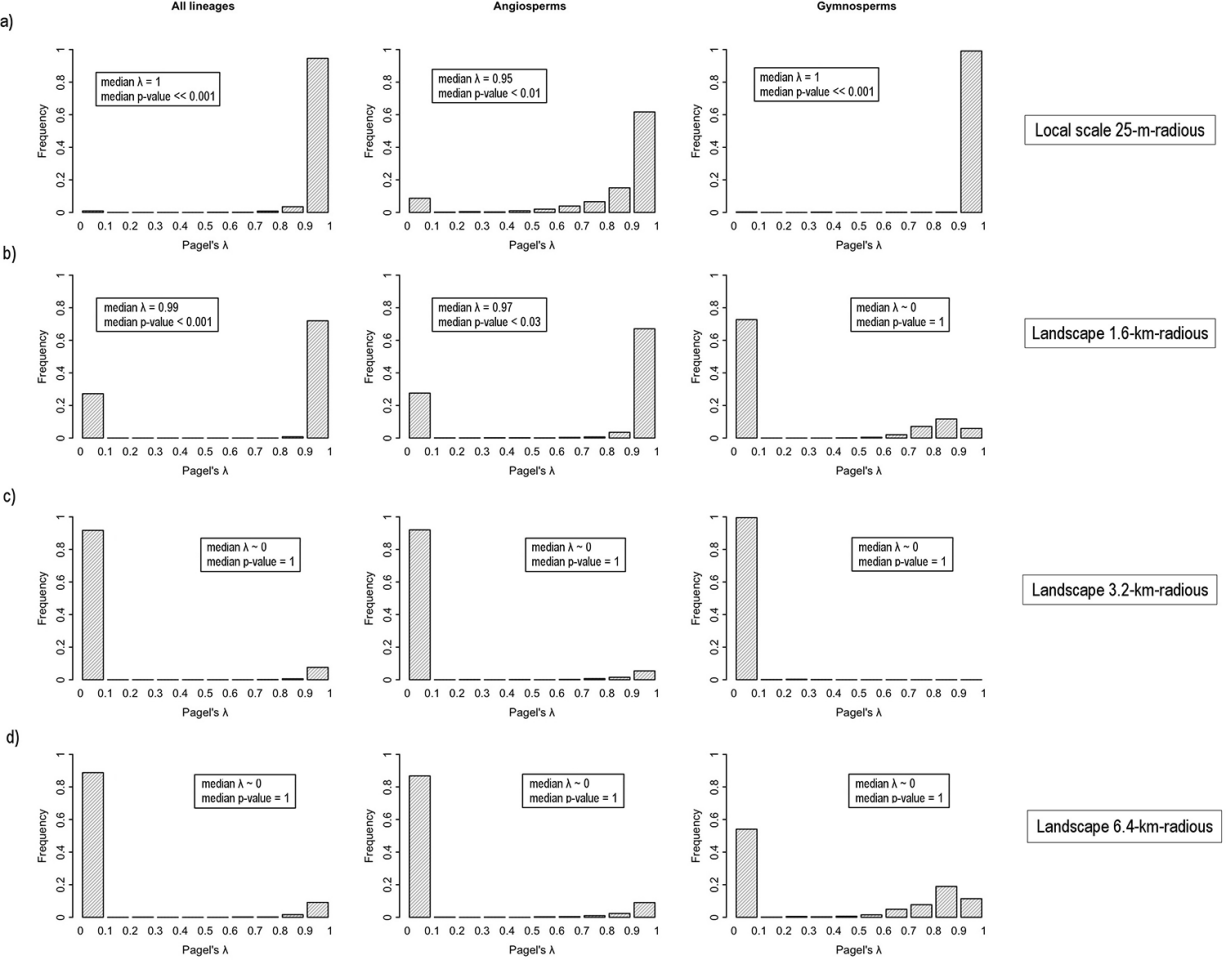
Phylogenetic signal in species response to forest canopy cover (Ω values) at different spatial scales. (a) Plot-level analyses (local scale). The histograms show the distribution of the λ statistic that resulted from analysing *n* = 1000 bootstrapped traits (i.e. Ω values within 95% CI, see text). Values close to 1 indicate that the trait evolved following a Brownian motion model of evolution (i.e. high phylogenetic signal), whereas close to zero values indicate lack of phylogenetic signal. The analyses were conducted using all the species in the study and angiosperms and gymnosperms separately. The overall phylogenetic signal (λ statistic) and statistical significance (*p*-value) are based on the median value of *n* = 1000 iterations. (b-d) Analyses conducted at different landscape scales.

Overall, we found a strong phylogenetic signal in species response to local forest loss (Fig 5a). When all lineages were analysed together, the median phylogenetic signal λ was equal to 1 (median *p*-value << 0.001). The pattern was similar within the gymnosperms (median λ = 1, median *p*-value << 0.001), and slightly weaker within the angiosperm lineage (median λ = 0.95, median *p*-value < 0.01). At the smallest landscape scale (1.6-km-radius), phylogenetic signal was also strong and significant for all lineages analysed together (median λ = 0.99, median *p*-value < 0.001) and the angiosperms (median λ = 0.97, median *p*-value < 0.03) (Fig 5b). However, phylogenetic signal was very weak and non-significant for the gymnosperms (median λ ~ 0, median *p*-value = 1; Fig 5b), and it almost entirely disappeared in all cases at intermediate (3.2-km-radius) and large (6.4-km-radius) landscape scales (Fig 5c and 5d).

## Discussion

The combinations of functional traits that modulate species responses to novel deforestation pressures might be evolutionarily conserved [26], and thus phylogenetic relationships may predict species survival in an increasingly human-deforested world [1]. Using the third Spanish National Forest Inventory as a case study, we found a strong phylogenetic signal in the response of tree species to forest loss at local and small landscape scales (Fig 5), suggesting that close relatives respond similarly to deforestation pressures.

Most species within the families Fagaceae, Pinaceae and Oleaceae (excluding *Phillyrea latifolia*) respectively showed a negative and similar response to decreased canopy cover at the plot level (Fig 4). As such, one might conclude that tree diversity in forest stands including many representatives of these lineages would be strongly affected by forest loss (i.e. most species would show a negative response to forest canopy cover reduction) as opposed to more phylogenetically diverse forests, which might ensure contrasted responses of the constituent species and thus less abrupt reductions in their abundances. This result highlights the importance of preserving high levels of phylogenetic diversity as a “natural insurance” for ecosystems [53], and may provide a foundational basis to inform conservation planning. Nevertheless, further research conducted across multiple biomes, lineages and trophic levels is required to confirm the generality of our results.

We found that the probability of occurrence of many species in the Fagaceae (i.e. *Quercus spp*.) decreased along with forest cover reduction across all the scales analysed (i.e. they are more prone to occur at high canopy cover irrespective of the scale, Fig 1a). Indeed, Fagaceae species (i.e. *Quercus ilex*, *Q*. *pyrenaica*, *Q*. *faginea*, *Q*. *suber*) constitute widespread and dense forest stands under natural conditions in the western Mediterranean [54, 35], the holm oak (*Q*. *ilex*) being the commonest in peninsular Spain. A substantial fraction of *Q*. *ilex* forests (and *Q*. *pyrenaica* and *Q*. *suber* to a minor extent) are transformed by humans into savanna-like systems (i.e. selective tree clearing) devoted primarily to livestock raising, the so-called Spanish dehesas [55]. However, despite the socio-economic importance of these semi-natural agroforestry ecosystems [56], dehesas fail to naturally regenerate [57] because oak seedlings require a relatively high canopy closure to survive [58, 59], which might be the cause of the high vulnerability of *Quercus* species to forest loss.

On the other hand, species within the Pinaceae also showed a negative response to decreased forest canopy cover at the local scale (Fig 4). Both *Pinus* and *Quercus* lineages include evergreen sclerophyllous-leaved trees (although *Quercus* also includes deciduous species), they are generally restricted to temperate latitudes in the Northern Hemisphere or to mountainous areas at lower latitudes, and they tend to form dense forest stands [60]. Besides, *Pinus* species show a strong phylogenetic signal in fire-adapted traits [61], which suggests that functionally relevant traits may have evolved conservatively in this lineage, thus leading to a similar response to forest loss between close relatives (Fig 5a).

It is important to note that all the common species analysed (i.e. most species in the Fagaceae and the Pinaceae) had higher stem densities with increased forest canopy cover at the local scale (i.e. negative plot-level Ω values). Although we expected negative Ω values for those species that tend to be dominant in closed forests, differences in Ω among them still reflected contrasted responses to forest loss. For example, the cork oak (*Quercus suber*) showed the lowest Ω values within the Fagaceae (with the notable exception of *Quercus canariensis*, a rare species in the study area) (Fig 4 and S1 Fig), and as such it tends to form extensive closed forests, notably in the southern half of the study area (e.g. Los Alcornocales Natural Park is characterized by the most extensive forest of cork oak in Spain, and one of the largest in the world). In contrast, the holm oak (*Quercus ilex*) showed higher Ω values than *Q*. *suber* (Fig 4 and S1 Fig), likely because the former is more tolerant to drought and cold than the latter [62], and therefore more prone to occur in open habitats.

The realm of positive Ω values and, consequently, of positive responses to local forest loss corresponds to species that tend to occur in open habitats and might be also present in closed forests, but not as dominant species. This was the case of most species in the Rosaceae (i.e. *Sorbus aucuparia*, *S*. *aria*, *Malus sylvestris* and *Crataegus monogyna*), which overall tended to show positive Ω values (Fig 4). *Sorbus aucuparia* and *S*. *aria* often occur at the edges of Atlantic forests in northern Spain [63], *Malus sylvestris* also occurs at the edges of wet forests or in open habitats due to its weak competitiveness and high light requirement [64], and *C*. *monogyna* is one of the most characteristic species of early-successional prickly shrub communities [35, 65]. These communities are critical for the natural regeneration of many forest types of peninsular Spain [66, 67], since they keep herbivores at bay from regeneration stands [68] and serve as the main microsites for the recruitment of many tree species [69, 70].

On the other hand, dispersal mode may also explain the trend towards positive Ω values in the Rosaceae [7]. Most species in this family possess fleshy fruits and are largely dispersed by animals [67], which may confer robustness to forest loss due to increased colonisation ability of open habitats [71, 72]. However, forest loss may still reduce colonisation ability of animal-dispersed trees if their main seed dispersers are forest-affiliated species. For example, Santos and Tellería [73] found that the abundance of *Turdus spp*., the main seed dispersers of the Spanish juniper (*J*. *thurifera*), was up to six times higher within large forest fragments, where the proportion of thrush pellets with intact seeds and seedling abundance of *J*. *thurifera* was also notably higher. Moreover, they found that seed consumption by *Apodemus sylvaticus* (a seed eater rodent) was up to nine times lower within large forest fragments. These results may largely explain the trend of *J*. *thurifera* to grow in extensively forested areas (Fig 4). Similar evidence has been reported for holm oak woodlands (*Quercus ilex*), where the balance between acorn consumption by wood mice (*Apodemus sylvaticus*) and effective dispersion (largely driven by the Eurasian jay, *Garrulus glandarius*) seems to depend on stem density [74, 75]. Together these findings highlight the impact that forest loss can have on species interaction networks [2].

Phylogenetic signal in species response to forest loss differed between angiosperms and gymnosperms, particularly at the small (1.6-km-radius) landscape scale (Fig 5). This suggests that despite we observed an overall phylogenetic signal in the data at local and small landscape scales (Fig 5a and 5b), a homogeneous rate of evolution (i.e. a single Brownian motion model for the entire phylogeny) may not be sufficient to explain evolutionary trajectories in species response to forest loss, as expected in phylogenetic trees involving many distant relative lineages [76]. Therefore, more inclusive studies including hundreds of species from multiple lineages and heterogeneous rates of evolution may be required to accurately model the evolution of species response to forest loss.

The presence of 76% of the species analysed in the study was best explained by forest canopy cover at local and small landscape scales. Further, the phylogenetic signal in species response was particularly strong at such scales, whereas it almost entirely disappeared at intermediate and large landscape scales (Fig 5). This suggests that the environmental drivers of species response to forest loss operate primarily at reduced spatial scales. Yet, a few pairs of closely related species such as *Betula pendula* and *Alnus glutinosa*, *Q*. *pubescens* and *Q*. *robur*, *Populus alba* and *P*. *nigra* showed similar positive responses at intermediate and large landscape scales (Fig 4 and S1 Fig). Generally, species with positive responses at the landscape scales had a negative response to local forest cover reduction, suggesting that they tend to grow in isolated forest patches (Fig 1c). It is noticeable that *A*. *glutinosa*, *P*. *alba* and *P*. *nigra* are characteristic of riparian forests, which may largely explain their tendency to grow in fragmented landscapes since riparian forest are often surrounded by highly deforested areas [77]. On the other hand, *Q*. *robur* is the most characteristic species in low-elevation forest stands in northern Spain, which are nowadays reduced to small and isolated fragments due to anthropogenic activities. This result highlights the importance of incorporating human-induced landscape transformations into ecological models to better understand current species distributions [78].

Finally, it is worthy to mention that some *Pinus* species such as *P*. *pinea* and *P*. *halepensis* also showed a contrasted response to forest loss between local and landscape scales (i.e. negative and positive responses, respectively; Fig 4 and S1 Fig), suggesting that they are prone to occur in isolated forest patches (Fig 1c). Consistent with this, *P*. *halepensis* is a fast-growing tree able to quickly colonize open and disturbed areas such as burned sites [79], which may explain its positive response to low forest cover at the landscape scale. On the other hand, *P*. *pinea* forms extensive forest stands along the coastline in southwestern and northeastern peninsular Spain that are embedded in a predominantly agricultural matrix. Besides, plots located along the coastline will necessarily show a low canopy cover in their surrounding areas due to the strong ecotone between land and sea areas.

## Conclusions

Identifying species sensitivity syndromes to deforestation is of outstanding importance if we are to preserve forest biodiversity in an increasingly deforested world [1]. Yet, evidence for the syndromes is often weak [19] or even contradictory (see [12] and references therein). We propose that available phylogenetic information could be used as a complementary reference to functional-based approaches for devising species vulnerability to forest loss based on ancestor-descendant relationships, and may thus provide a foundational basis to inform conservation planning. Importantly, our approach may be particularly valuable to analyse poorly characterized taxa for which functional information is largely missing [80, 18, 19].

Using the third Spanish National Forest Inventory as a case study, we have shown how evolutionary history largely determines the response of tree species to forest loss, particularly at reduced spatial scales. As such, forest stands with low levels of phylogenetic diversity would be comprised of species that are expected to show similar responses to deforestation pressures (and therefore there is a high probability for a general negative response), whereas phylogenetically diverse forests would respond less abruptly, since the latter would comprise a mix of species with contrasted responses. Although we focused on tree species, our results could be extended to other taxonomic groups whose distribution is largely determined by the presence of forest stands [81], and highlight the importance of preserving high levels of phylogenetic diversity as a “natural insurance” for ecosystems [53].

## Supporting information

S1 Table Fossil information and minimum and maximum age constraints used for the dating analysis. Fossils were assigned to the most recent common ancestors (MRCA) of the listed taxa.
(XLSX)

S1 Fig Response of tree species (Ω values) to forest canopy cover measured at different landscape scales (concentric circles centred on the plots with radiuses of 1.6, 3.2 and 6.4 km-radius, respectively). The bars and dots correspond to 95% CI of Ω values. The response of species was considered negative when the 95% CI of Ω completely laid below zero (red colour), positive when the 95% CI completely laid above zero (green colour), and neutral if the 95% CI included the zero (grey colour). The gaps in the figure represent those cases where the Poisson models failed to explain the probability of occurrences of species (see text). The scale bar in the phylogeny represents millions of years.
(DOC)
