# Supplementary figures and images for: Evolutionary history predicts the response of tree species to forest loss: A case study in peninsular Spain
Source: PLoS One. 2018 Sep 20;13(9):e0204365. doi: 10.1371/journal.pone.0204365 (PMC6147707; doi:10.1371/journal.pone.0204365)

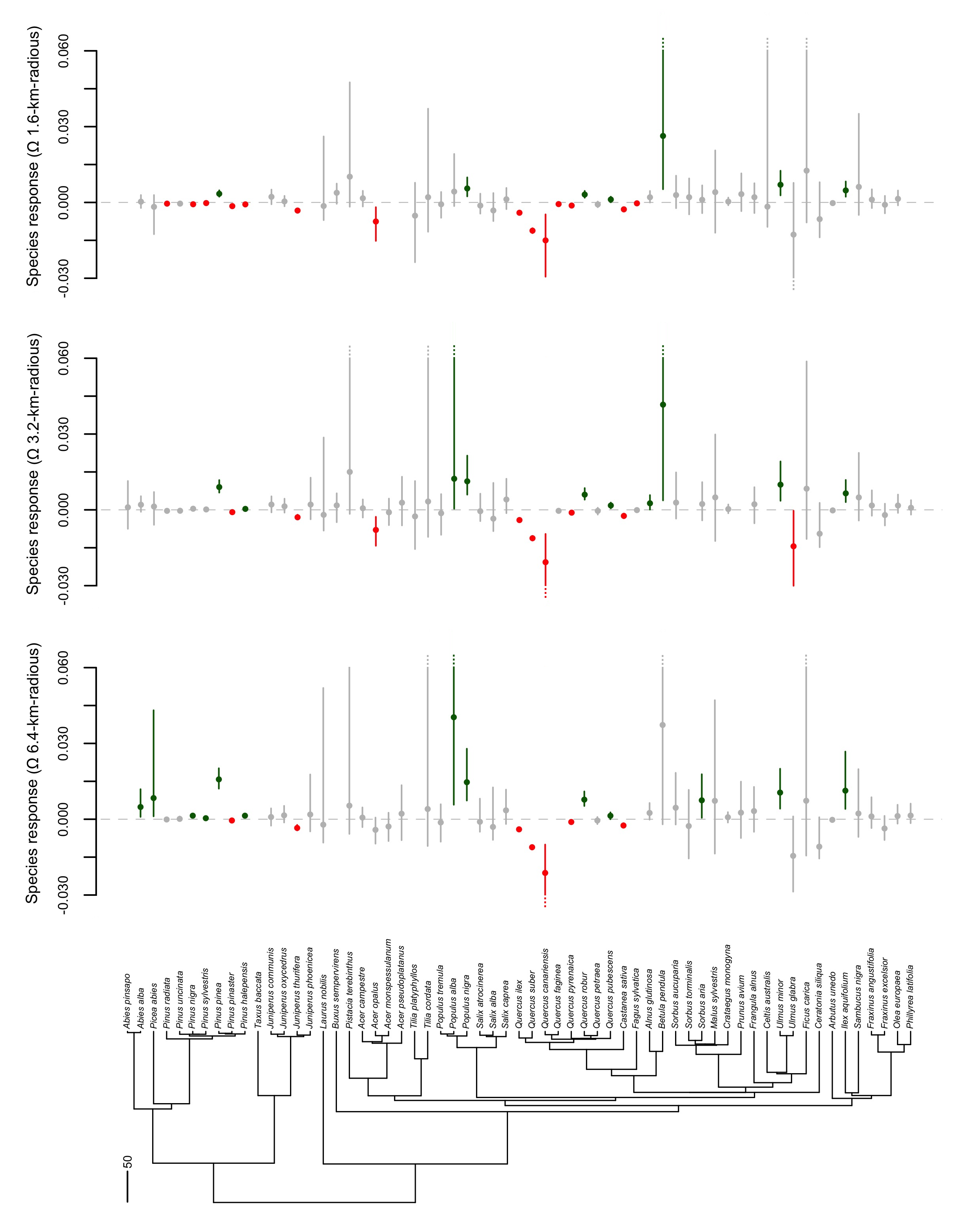

Supplement: S1 Fig — The bars and dots correspond to 95% CI of Ω values. The response of species was considered negative when the 95% CI of Ω completely laid below zero (red colour), positive when the 95% CI completely laid above zero (green colour), and neutral if the 95% CI included the zero (grey colour). The gaps in the figure represent those cases where the Poisson models failed to explain the probability of occurrences of species (see text). The scale bar in the phylogeny represents millions of years. (DOC) [file pone.0204365.s002.doc]
